# 3-Nitro-*N*-(8-quinol­yl)benzamide

**DOI:** 10.1107/S1600536808037811

**Published:** 2008-11-20

**Authors:** Gang Lei, Lin-Hai Jing, Li Zhou

**Affiliations:** a School of Chemistry and Chemical Engineering, China West Normal University, Nanchong 637002, People’s Republic of China

## Abstract

The title compound, C_16_H_11_N_3_O_3_, crystallizes with two independent mol­ecules which are almost identical to each other in the asymmetric unit. The dihedral angle between the quinoline ring system and the nitro­benzene ring is 51.04 (9)° in one of the mol­ecules and 48.91 (9)° in the other. The crystal packing is stabilized by C—H⋯O hydrogen bonds and π–π inter­actions, with a centroid–centroid distance of 3.6010 (15) Å.

## Related literature

For general background, see: Oku *et al.* (1998 ▶, 1999 ▶). For a related structure, see: Lei *et al.* (2008 ▶).
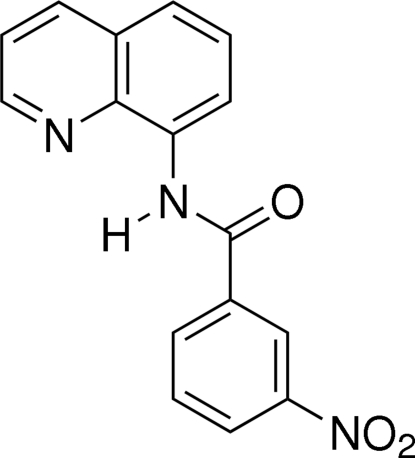


## Experimental

### 

#### Crystal data

C_16_H_11_N_3_O_3_
*M* *_r_* = 293.28
Monoclinic, 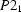

*a* = 7.3783 (14) Å
*b* = 23.878 (5) Å
*c* = 7.4371 (14) Å
β = 90.775 (3)°
*V* = 1310.2 (4) Å^3^
*Z* = 4
Mo *K*α radiation
μ = 0.11 mm^−1^
*T* = 93 (2) K
0.40 × 0.30 × 0.23 mm

#### Data collection

Rigaku R-AXIS RAPID diffractometer
Absorption correction: none
10838 measured reflections
3071 independent reflections
2860 reflections with *I* > 2σ(*I*)
*R*_int_ = 0.030

#### Refinement

*R*[*F* ^2^ > 2σ(*F* ^2^)] = 0.037
*wR*(*F* ^2^) = 0.075
*S* = 1.14
3071 reflections
397 parameters
1 restraint
H-atom parameters constrained
Δρ_max_ = 0.19 e Å^−3^
Δρ_min_ = −0.19 e Å^−3^

### 

Data collection: *RAPID-AUTO* (Rigaku, 2004 ▶); cell refinement: *RAPID-AUTO*; data reduction: *RAPID-AUTO*; program(s) used to solve structure: *SHELXS97* (Sheldrick, 2008 ▶); program(s) used to refine structure: *SHELXL97* (Sheldrick, 2008 ▶); molecular graphics: *XP* in *SHELXTL* (Sheldrick, 2008 ▶); software used to prepare material for publication: *SHELXL97*.

## Supplementary Material

Crystal structure: contains datablocks global, I. DOI: 10.1107/S1600536808037811/ci2717sup1.cif

Structure factors: contains datablocks I. DOI: 10.1107/S1600536808037811/ci2717Isup2.hkl

Additional supplementary materials:  crystallographic information; 3D view; checkCIF report

## Figures and Tables

**Table 1:** Hydrogen-bond geometry (Å, °)

*D*—H⋯*A*	*D*—H	H⋯*A*	*D*⋯*A*	*D*—H⋯*A*
C2—H2⋯O2^i^	0.95	2.56	3.395 (3)	147
C2*A*—H2*A*⋯O2^i^	0.95	2.50	3.346 (3)	148
C4—H4⋯O2*A*^i^	0.95	2.48	3.317 (3)	146
C16—H16⋯O3*A*^ii^	0.95	2.46	3.147 (3)	130
C16*A*—H16*A*⋯O1^iii^	0.95	2.50	3.198 (3)	130
C17—H17⋯O1*A*^iv^	0.95	2.45	3.396 (3)	172

Symmetry codes: (i) 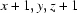; (ii) 
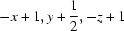
; (iii) 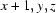; (iv) 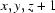.

**Fig. 1.**
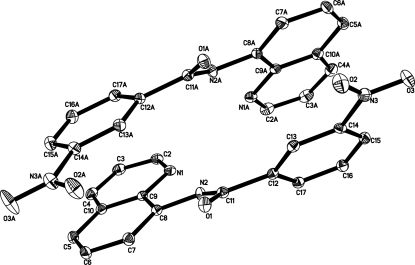
The asymmetric unit of the title compound, showing two independent molecules. Displacement ellipsoids are drawn at the 50% probability level. H atoms have been omitted for clarity.

## supplementary crystallographic information

### Comment

Quinoline derivatives are important compounds for the treatment of bone metabolic disorders (Oku *et al.*,  1998) and as H^+^-ATPases inhibitors (Oku *et al.*,  1999). Previously, we have reported the crystal structure of (2-nitrophenyl)-N-(8-quinolyl)carboxamide (Lei *et al.*,  2008). Now, we report here the crystal structure of the title compound.

The asymmetric unit of the title compound contains two independent molecules (Fig. 1) almost identical to each other in structure. Bond lengths and angles of the two molecules agree with each other and are comparable to those observed for (2-nitrophenyl)-N-(8-quinolyl)carboxamide (Lei *et al.*, 2008). The quinoline ring systems are planar, with a maximum deviation of 0.017 (5) Å for atom C8 and 0.008 (5) Å for atom C9A, respectively. As a result of steric effects, the amide groups are twisted away from the planes of the quinoline benzene rings and 2-nitro substituted benzene rings (Fig. 1). The C5-C10 and C12-C17 planes form dihedral angles of 18.67 (1) and 32.89 (1)°, respectively, with the O1/N2/C8/C11 plane. Similarly, the C5A—C10A and C12A—C17A planes form dihedral angles of 20.90 (1) and 28.46 (1)°, respectively, with the O1A/N2A/C8A/C11A plane. The dihedral angle between C12-C17 and O2/O3/N3/C14 planes is 1.07 (1)° and that between C12A—C17A and O2A/O3A/N3A/C14A planes is 2.28 (1)°. The dihedral angle between quinoline ring system and nitrobenzene ring is 51.04 (9)° in one of the molecules and 48.91 (9)° in the other (with suffix A).

The crystal packing is stabilized by C—H···O hydrogen bonds (Table 1) and π-π interactionS involving C5A—C10A (centroid *Cg*1) and C12—C17 (centroid *Cg*2) benzene rings, with a *Cg*1···*Cg*2 distance of 3.6010 (15) Å.

### Experimental

*m*-Nitrobenzoic acid (2 mmol) and an excess of thionyl chloride (3 mmol) in dioxane (20 ml) were boiled under reflux for 6 h. The solution was distilled under reduced pressure and a yellow solid was obtained. 8-Aminoquinoline (2 mmol) in tetrahydrofuran (20 ml) was added to the yellow solid and boiled under reflux for 6 h. The solution was then cooled to ambient temperature and filtered to remove the tetrahydrofuran. The precipitate was dissolved in dimethyl sulfoxide and the solution was allowed to stand for one month at ambient temperature, after which time white single crystals of the title compound suitable for X-ray diffraction were obtained.

### Refinement

All H atoms were placed in calculated positions, with C-H = 0.95 Å and N-H = 0.88 Å, and refined using a riding model, with *U*_iso_(H) = 1.2*U*_eq_(C,N). In the absence of significant anomalous scattering, Friedel pairs were merged prior to the final refinement.

### Crystal data

**Table:**

C_16_H_11_N_3_O_3_	*F*_000_ = 608
*M**_r_* = 293.28	*D*_x_ = 1.487 Mg m^−^^3^
Monoclinic, *P*2_1_	Mo *K*α radiation λ = 0.71073 Å
Hall symbol: P 2yb	Cell parameters from 4482 reflections
*a* = 7.3783 (14) Å	θ = 3.2–27.5º
*b* = 23.878 (5) Å	µ = 0.11 mm^−^^1^
*c* = 7.4371 (14) Å	*T* = 93 (2) K
β = 90.775 (3)º	Block, white
*V* = 1310.2 (4) Å^3^	0.40 × 0.30 × 0.23 mm
*Z* = 4	

### Data collection

**Table:**

Rigaku R-AXIS RAPID diffractometer	2860 reflections with *I* > 2σ(*I*)
Radiation source: fine-focus sealed tube	*R*_int_ = 0.030
Monochromator: graphite	θ_max_ = 27.5º
*T* = 93(2) K	θ_min_ = 3.2º
ω scans	*h* = −9→9
Absorption correction: none	*k* = −31→30
10838 measured reflections	*l* = −9→9
3071 independent reflections	

### Refinement

**Table:**

Refinement on *F*^2^	Secondary atom site location: difference Fourier map
Least-squares matrix: full	Hydrogen site location: inferred from neighbouring sites
*R*[*F*^2^ > 2σ(*F*^2^)] = 0.037	H-atom parameters constrained
*wR*(*F*^2^) = 0.075	*w* = 1/[σ^2^(*F*_o_^2^) + (0.0354*P*)^2^] where *P* = (*F*_o_^2^ + 2*F*_c_^2^)/3
*S* = 1.14	(Δ/σ)_max_ = 0.001
3071 reflections	Δρ_max_ = 0.19 e Å^−^^3^
397 parameters	Δρ_min_ = −0.19 e Å^−^^3^
1 restraint	Extinction correction: none
Primary atom site location: structure-invariant direct methods	

### Special details

**Table:**

Geometry. All e.s.d.'s (except the e.s.d. in the dihedral angle between two l.s. planes) are estimated using the full covariance matrix. The cell e.s.d.'s are taken into account individually in the estimation of e.s.d.'s in distances, angles and torsion angles; correlations between e.s.d.'s in cell parameters are only used when they are defined by crystal symmetry. An approximate (isotropic) treatment of cell e.s.d.'s is used for estimating e.s.d.'s involving l.s. planes.
Refinement. Refinement of *F*^2^ against ALL reflections. The weighted *R*-factor *wR* and goodness of fit *S* are based on *F*^2^, conventional *R*-factors *R* are based on *F*, with *F* set to zero for negative *F*^2^. The threshold expression of *F*^2^ > σ(*F*^2^) is used only for calculating *R*-factors(gt) *etc*. and is not relevant to the choice of reflections for refinement. *R*-factors based on *F*^2^ are statistically about twice as large as those based on *F*, and *R*- factors based on ALL data will be even larger.

### Fractional atomic coordinates and isotropic or equivalent isotropic displacement parameters (Å^2^)

**Table:**

	*x*	*y*	*z*	*U*_iso_*/*U*_eq_	
O1	0.3802 (2)	0.19096 (7)	0.4467 (2)	0.0229 (4)	
O2	0.1871 (3)	0.35241 (8)	0.0594 (2)	0.0334 (4)	
O3	0.1573 (2)	0.43422 (7)	0.1784 (2)	0.0308 (4)	
N1	0.9400 (3)	0.22508 (8)	0.7724 (3)	0.0202 (4)	
N2	0.6102 (3)	0.21775 (8)	0.6364 (3)	0.0195 (4)	
H2N	0.6600	0.2477	0.6850	0.023*	
N3	0.2028 (3)	0.38491 (8)	0.1864 (3)	0.0220 (4)	
C2	1.1064 (3)	0.23028 (11)	0.8387 (3)	0.0233 (5)	
H2	1.1537	0.2670	0.8546	0.028*	
C3	1.2169 (3)	0.18492 (11)	0.8868 (3)	0.0263 (5)	
H3	1.3365	0.1910	0.9315	0.032*	
C4	1.1508 (3)	0.13180 (11)	0.8688 (3)	0.0255 (5)	
H4	1.2237	0.1006	0.9024	0.031*	
C5	0.8918 (3)	0.07037 (10)	0.7766 (3)	0.0224 (5)	
H5	0.9567	0.0374	0.8082	0.027*	
C6	0.7193 (3)	0.06662 (10)	0.7085 (3)	0.0237 (5)	
H6	0.6655	0.0307	0.6949	0.028*	
C7	0.6189 (3)	0.11439 (10)	0.6579 (3)	0.0218 (5)	
H7	0.4997	0.1105	0.6097	0.026*	
C8	0.6946 (3)	0.16665 (9)	0.6785 (3)	0.0181 (5)	
C9	0.8739 (3)	0.17209 (9)	0.7526 (3)	0.0181 (5)	
C10	0.9734 (3)	0.12345 (10)	0.7999 (3)	0.0206 (5)	
C11	0.4622 (3)	0.22707 (10)	0.5310 (3)	0.0184 (5)	
C12	0.4060 (3)	0.28758 (9)	0.5227 (3)	0.0165 (5)	
C13	0.3286 (3)	0.30764 (9)	0.3644 (3)	0.0178 (5)	
H13	0.3102	0.2836	0.2640	0.021*	
C14	0.2792 (3)	0.36325 (9)	0.3560 (3)	0.0169 (4)	
C15	0.3002 (3)	0.39973 (10)	0.4987 (3)	0.0205 (5)	
H15	0.2654	0.4379	0.4883	0.025*	
C16	0.3735 (3)	0.37886 (10)	0.6575 (3)	0.0207 (5)	
H16	0.3869	0.4027	0.7591	0.025*	
C17	0.4276 (3)	0.32341 (10)	0.6691 (3)	0.0188 (5)	
H17	0.4798	0.3097	0.7779	0.023*	
O1A	0.6075 (2)	0.26160 (7)	0.0420 (2)	0.0221 (4)	
O2A	0.4490 (2)	0.06211 (8)	0.1206 (3)	0.0371 (5)	
O3A	0.6518 (3)	0.00459 (8)	0.2183 (3)	0.0488 (6)	
N1A	0.8655 (3)	0.35147 (8)	0.5612 (2)	0.0213 (4)	
N2A	0.7920 (3)	0.29510 (8)	0.2650 (3)	0.0189 (4)	
H2NA	0.8755	0.2849	0.3440	0.023*	
N3A	0.6014 (3)	0.05199 (8)	0.1811 (3)	0.0268 (5)	
C2A	0.9074 (3)	0.37849 (11)	0.7114 (3)	0.0267 (6)	
H2A	0.9586	0.3578	0.8086	0.032*	
C3A	0.8798 (3)	0.43636 (12)	0.7346 (3)	0.0299 (6)	
H3A	0.9132	0.4540	0.8446	0.036*	
C4A	0.8049 (3)	0.46662 (11)	0.5980 (3)	0.0273 (6)	
H4A	0.7831	0.5055	0.6130	0.033*	
C5A	0.6826 (3)	0.46776 (10)	0.2831 (3)	0.0241 (5)	
H5A	0.6603	0.5069	0.2881	0.029*	
C6A	0.6403 (3)	0.43863 (10)	0.1308 (3)	0.0235 (5)	
H6A	0.5880	0.4578	0.0309	0.028*	
C7A	0.6729 (3)	0.38034 (10)	0.1186 (3)	0.0217 (5)	
H7A	0.6420	0.3606	0.0115	0.026*	
C8A	0.7486 (3)	0.35259 (9)	0.2608 (3)	0.0174 (5)	
C9A	0.7935 (3)	0.38198 (10)	0.4228 (3)	0.0185 (5)	
C10A	0.7595 (3)	0.44004 (10)	0.4334 (3)	0.0211 (5)	
C11A	0.7202 (3)	0.25366 (10)	0.1617 (3)	0.0185 (5)	
C12A	0.7870 (3)	0.19612 (10)	0.2061 (3)	0.0174 (5)	
C13A	0.6706 (3)	0.15156 (10)	0.1700 (3)	0.0189 (5)	
H13A	0.5544	0.1578	0.1174	0.023*	
C14A	0.7274 (3)	0.09834 (10)	0.2121 (3)	0.0194 (5)	
C15A	0.8969 (3)	0.08665 (10)	0.2844 (3)	0.0231 (5)	
H15A	0.9326	0.0492	0.3099	0.028*	
C16A	1.0131 (3)	0.13095 (10)	0.3186 (3)	0.0226 (5)	
H16A	1.1307	0.1242	0.3674	0.027*	
C17A	0.9574 (3)	0.18545 (10)	0.2814 (3)	0.0204 (5)	
H17A	1.0366	0.2158	0.3077	0.025*	

### Atomic displacement parameters (Å^2^)

**Table:**

	*U*^11^	*U*^22^	*U*^33^	*U*^12^	*U*^13^	*U*^23^
O1	0.0230 (9)	0.0215 (9)	0.0242 (9)	0.0012 (7)	−0.0047 (7)	−0.0039 (7)
O2	0.0457 (11)	0.0319 (10)	0.0223 (9)	0.0074 (9)	−0.0102 (8)	−0.0034 (8)
O3	0.0382 (11)	0.0207 (9)	0.0333 (10)	0.0058 (8)	−0.0072 (8)	0.0065 (8)
N1	0.0173 (10)	0.0224 (10)	0.0209 (10)	−0.0028 (8)	0.0014 (8)	0.0017 (9)
N2	0.0215 (10)	0.0154 (10)	0.0214 (10)	0.0025 (8)	−0.0044 (8)	−0.0030 (8)
N3	0.0189 (10)	0.0249 (11)	0.0222 (10)	0.0011 (8)	−0.0008 (8)	0.0029 (9)
C2	0.0187 (11)	0.0264 (14)	0.0247 (13)	−0.0039 (10)	0.0010 (10)	0.0014 (11)
C3	0.0187 (12)	0.0359 (14)	0.0242 (13)	−0.0010 (11)	−0.0007 (10)	0.0016 (11)
C4	0.0209 (12)	0.0329 (14)	0.0228 (12)	0.0065 (11)	0.0003 (10)	0.0021 (11)
C5	0.0242 (12)	0.0197 (12)	0.0233 (12)	0.0043 (10)	0.0008 (10)	0.0020 (10)
C6	0.0298 (13)	0.0166 (12)	0.0248 (12)	−0.0005 (10)	0.0026 (10)	−0.0013 (10)
C7	0.0222 (12)	0.0198 (12)	0.0233 (12)	−0.0008 (9)	−0.0003 (10)	−0.0022 (10)
C8	0.0201 (11)	0.0178 (11)	0.0165 (11)	0.0016 (9)	0.0027 (9)	0.0005 (9)
C9	0.0206 (11)	0.0185 (12)	0.0152 (11)	0.0017 (9)	0.0038 (9)	0.0004 (9)
C10	0.0212 (12)	0.0233 (12)	0.0175 (12)	0.0029 (10)	0.0023 (9)	0.0014 (10)
C11	0.0191 (11)	0.0190 (11)	0.0170 (11)	−0.0009 (9)	0.0010 (9)	−0.0002 (10)
C12	0.0125 (10)	0.0180 (11)	0.0188 (11)	−0.0009 (9)	−0.0006 (9)	−0.0001 (9)
C13	0.0144 (10)	0.0196 (11)	0.0195 (11)	−0.0028 (9)	0.0003 (9)	−0.0022 (9)
C14	0.0135 (10)	0.0207 (11)	0.0166 (11)	−0.0022 (8)	−0.0011 (8)	0.0043 (9)
C15	0.0185 (11)	0.0177 (12)	0.0252 (13)	0.0001 (9)	0.0023 (10)	0.0009 (10)
C16	0.0224 (12)	0.0186 (12)	0.0212 (12)	−0.0022 (9)	0.0009 (10)	−0.0022 (10)
C17	0.0164 (11)	0.0219 (12)	0.0181 (12)	−0.0006 (9)	0.0000 (9)	0.0002 (9)
O1A	0.0235 (8)	0.0199 (8)	0.0229 (9)	0.0015 (7)	−0.0048 (7)	0.0002 (7)
O2A	0.0302 (11)	0.0256 (10)	0.0551 (12)	−0.0065 (8)	−0.0173 (9)	0.0057 (9)
O3A	0.0509 (14)	0.0158 (9)	0.0787 (15)	−0.0034 (9)	−0.0272 (12)	0.0107 (10)
N1A	0.0175 (10)	0.0261 (11)	0.0203 (10)	−0.0014 (8)	−0.0005 (8)	0.0019 (8)
N2A	0.0182 (10)	0.0166 (10)	0.0216 (10)	0.0010 (8)	−0.0046 (8)	0.0015 (8)
N3A	0.0304 (12)	0.0208 (11)	0.0290 (12)	−0.0007 (9)	−0.0063 (10)	0.0023 (9)
C2A	0.0210 (12)	0.0384 (15)	0.0206 (12)	−0.0023 (11)	−0.0020 (10)	−0.0013 (11)
C3A	0.0277 (14)	0.0364 (15)	0.0257 (13)	−0.0091 (12)	0.0030 (11)	−0.0106 (12)
C4A	0.0229 (13)	0.0242 (13)	0.0347 (14)	−0.0054 (10)	0.0047 (11)	−0.0061 (11)
C5A	0.0189 (11)	0.0170 (12)	0.0365 (14)	−0.0011 (9)	0.0040 (10)	0.0045 (10)
C6A	0.0217 (12)	0.0204 (12)	0.0284 (13)	0.0013 (10)	−0.0020 (10)	0.0076 (11)
C7A	0.0214 (11)	0.0214 (12)	0.0224 (12)	−0.0009 (9)	0.0001 (10)	0.0022 (10)
C8A	0.0158 (10)	0.0154 (11)	0.0210 (12)	−0.0009 (9)	0.0019 (9)	0.0012 (9)
C9A	0.0144 (10)	0.0198 (11)	0.0214 (11)	−0.0024 (9)	0.0027 (9)	0.0017 (9)
C10A	0.0171 (11)	0.0203 (12)	0.0260 (12)	−0.0032 (9)	0.0029 (9)	−0.0026 (10)
C11A	0.0173 (11)	0.0199 (12)	0.0183 (12)	0.0005 (9)	0.0012 (10)	−0.0001 (10)
C12A	0.0192 (11)	0.0165 (11)	0.0164 (11)	0.0001 (9)	0.0015 (9)	0.0005 (9)
C13A	0.0224 (12)	0.0203 (12)	0.0138 (11)	0.0016 (9)	−0.0022 (9)	0.0010 (9)
C14A	0.0206 (11)	0.0188 (12)	0.0188 (11)	−0.0016 (9)	−0.0018 (9)	0.0003 (9)
C15A	0.0258 (12)	0.0196 (12)	0.0236 (13)	0.0023 (10)	−0.0026 (10)	−0.0004 (10)
C16A	0.0184 (12)	0.0242 (13)	0.0251 (12)	0.0030 (10)	−0.0038 (10)	0.0006 (10)
C17A	0.0184 (11)	0.0218 (12)	0.0211 (11)	−0.0010 (9)	0.0001 (9)	−0.0006 (10)

### Geometric parameters (Å, °)

**Table:**

O1—C11	1.222 (3)	O1A—C11A	1.224 (3)
O2—N3	1.227 (2)	O2A—N3A	1.230 (3)
O3—N3	1.225 (3)	O3A—N3A	1.222 (3)
N1—C2	1.323 (3)	N1A—C2A	1.323 (3)
N1—C9	1.363 (3)	N1A—C9A	1.363 (3)
N2—C11	1.354 (3)	N2A—C11A	1.356 (3)
N2—C8	1.403 (3)	N2A—C8A	1.410 (3)
N2—H2N	0.88	N2A—H2NA	0.88
N3—C14	1.469 (3)	N3A—C14A	1.462 (3)
C2—C3	1.399 (3)	C2A—C3A	1.408 (4)
C2—H2	0.95	C2A—H2A	0.95
C3—C4	1.365 (3)	C3A—C4A	1.358 (4)
C3—H3	0.95	C3A—H3A	0.95
C4—C10	1.414 (3)	C4A—C10A	1.415 (3)
C4—H4	0.95	C4A—H4A	0.95
C5—C6	1.366 (3)	C5A—C6A	1.362 (3)
C5—C10	1.413 (3)	C5A—C10A	1.412 (3)
C5—H5	0.95	C5A—H5A	0.95
C6—C7	1.409 (3)	C6A—C7A	1.416 (3)
C6—H6	0.95	C6A—H6A	0.95
C7—C8	1.375 (3)	C7A—C8A	1.361 (3)
C7—H7	0.95	C7A—H7A	0.95
C8—C9	1.432 (3)	C8A—C9A	1.429 (3)
C9—C10	1.416 (3)	C9A—C10A	1.411 (3)
C11—C12	1.504 (3)	C11A—C12A	1.495 (3)
C12—C13	1.387 (3)	C12A—C13A	1.391 (3)
C12—C17	1.392 (3)	C12A—C17A	1.393 (3)
C13—C14	1.378 (3)	C13A—C14A	1.373 (3)
C13—H13	0.95	C13A—H13A	0.95
C14—C15	1.380 (3)	C14A—C15A	1.383 (3)
C15—C16	1.385 (3)	C15A—C16A	1.383 (3)
C15—H15	0.95	C15A—H15A	0.95
C16—C17	1.385 (3)	C16A—C17A	1.391 (3)
C16—H16	0.95	C16A—H16A	0.95
C17—H17	0.95	C17A—H17A	0.95
			
C2—N1—C9	117.2 (2)	C2A—N1A—C9A	117.5 (2)
C11—N2—C8	128.63 (19)	C11A—N2A—C8A	127.67 (18)
C11—N2—H2N	115.7	C11A—N2A—H2NA	116.2
C8—N2—H2N	115.7	C8A—N2A—H2NA	116.2
O3—N3—O2	123.2 (2)	O3A—N3A—O2A	122.6 (2)
O3—N3—C14	118.80 (19)	O3A—N3A—C14A	118.3 (2)
O2—N3—C14	118.03 (19)	O2A—N3A—C14A	119.05 (19)
N1—C2—C3	123.9 (2)	N1A—C2A—C3A	123.3 (2)
N1—C2—H2	118.1	N1A—C2A—H2A	118.3
C3—C2—H2	118.1	C3A—C2A—H2A	118.3
C4—C3—C2	119.2 (2)	C4A—C3A—C2A	119.2 (2)
C4—C3—H3	120.4	C4A—C3A—H3A	120.4
C2—C3—H3	120.4	C2A—C3A—H3A	120.4
C3—C4—C10	119.6 (2)	C3A—C4A—C10A	119.8 (2)
C3—C4—H4	120.2	C3A—C4A—H4A	120.1
C10—C4—H4	120.2	C10A—C4A—H4A	120.1
C6—C5—C10	119.8 (2)	C6A—C5A—C10A	120.3 (2)
C6—C5—H5	120.1	C6A—C5A—H5A	119.9
C10—C5—H5	120.1	C10A—C5A—H5A	119.9
C5—C6—C7	122.0 (2)	C5A—C6A—C7A	121.2 (2)
C5—C6—H6	119.0	C5A—C6A—H6A	119.4
C7—C6—H6	119.0	C7A—C6A—H6A	119.4
C8—C7—C6	119.6 (2)	C8A—C7A—C6A	119.8 (2)
C8—C7—H7	120.2	C8A—C7A—H7A	120.1
C6—C7—H7	120.2	C6A—C7A—H7A	120.1
C7—C8—N2	125.8 (2)	C7A—C8A—N2A	125.6 (2)
C7—C8—C9	119.8 (2)	C7A—C8A—C9A	120.2 (2)
N2—C8—C9	114.33 (19)	N2A—C8A—C9A	114.17 (18)
N1—C9—C10	123.4 (2)	N1A—C9A—C10A	123.4 (2)
N1—C9—C8	116.94 (19)	N1A—C9A—C8A	117.2 (2)
C10—C9—C8	119.6 (2)	C10A—C9A—C8A	119.3 (2)
C5—C10—C4	124.2 (2)	C9A—C10A—C5A	119.1 (2)
C5—C10—C9	119.1 (2)	C9A—C10A—C4A	116.7 (2)
C4—C10—C9	116.7 (2)	C5A—C10A—C4A	124.3 (2)
O1—C11—N2	124.8 (2)	O1A—C11A—N2A	123.7 (2)
O1—C11—C12	121.5 (2)	O1A—C11A—C12A	121.4 (2)
N2—C11—C12	113.66 (19)	N2A—C11A—C12A	114.84 (19)
C13—C12—C17	119.5 (2)	C13A—C12A—C17A	119.3 (2)
C13—C12—C11	118.46 (19)	C13A—C12A—C11A	117.4 (2)
C17—C12—C11	122.01 (19)	C17A—C12A—C11A	123.4 (2)
C14—C13—C12	118.5 (2)	C14A—C13A—C12A	118.6 (2)
C14—C13—H13	120.8	C14A—C13A—H13A	120.7
C12—C13—H13	120.8	C12A—C13A—H13A	120.7
C15—C14—C13	123.1 (2)	C13A—C14A—C15A	123.2 (2)
C15—C14—N3	118.4 (2)	C13A—C14A—N3A	118.3 (2)
C13—C14—N3	118.4 (2)	C15A—C14A—N3A	118.6 (2)
C14—C15—C16	117.8 (2)	C14A—C15A—C16A	118.2 (2)
C14—C15—H15	121.1	C14A—C15A—H15A	120.9
C16—C15—H15	121.1	C16A—C15A—H15A	120.9
C17—C16—C15	120.4 (2)	C15A—C16A—C17A	119.9 (2)
C17—C16—H16	119.8	C15A—C16A—H16A	120.1
C15—C16—H16	119.8	C17A—C16A—H16A	120.1
C16—C17—C12	120.6 (2)	C16A—C17A—C12A	120.9 (2)
C16—C17—H17	119.7	C16A—C17A—H17A	119.5
C12—C17—H17	119.7	C12A—C17A—H17A	119.5
			
C9—N1—C2—C3	0.7 (3)	C9A—N1A—C2A—C3A	0.5 (3)
N1—C2—C3—C4	−1.3 (4)	N1A—C2A—C3A—C4A	0.7 (4)
C2—C3—C4—C10	0.8 (3)	C2A—C3A—C4A—C10A	−1.4 (4)
C10—C5—C6—C7	0.8 (4)	C10A—C5A—C6A—C7A	−0.4 (4)
C5—C6—C7—C8	−0.6 (4)	C5A—C6A—C7A—C8A	−0.3 (4)
C6—C7—C8—N2	−179.1 (2)	C6A—C7A—C8A—N2A	−178.4 (2)
C6—C7—C8—C9	−0.5 (3)	C6A—C7A—C8A—C9A	0.7 (3)
C11—N2—C8—C7	−17.4 (4)	C11A—N2A—C8A—C7A	−22.8 (4)
C11—N2—C8—C9	164.0 (2)	C11A—N2A—C8A—C9A	158.1 (2)
C2—N1—C9—C10	0.4 (3)	C2A—N1A—C9A—C10A	−1.0 (3)
C2—N1—C9—C8	−179.2 (2)	C2A—N1A—C9A—C8A	180.0 (2)
C7—C8—C9—N1	−178.9 (2)	C7A—C8A—C9A—N1A	178.7 (2)
N2—C8—C9—N1	−0.2 (3)	N2A—C8A—C9A—N1A	−2.1 (3)
C7—C8—C9—C10	1.5 (3)	C7A—C8A—C9A—C10A	−0.4 (3)
N2—C8—C9—C10	−179.8 (2)	N2A—C8A—C9A—C10A	178.8 (2)
C6—C5—C10—C4	−179.9 (2)	N1A—C9A—C10A—C5A	−179.3 (2)
C6—C5—C10—C9	0.2 (3)	C8A—C9A—C10A—C5A	−0.3 (3)
C3—C4—C10—C5	−179.7 (2)	N1A—C9A—C10A—C4A	0.3 (3)
C3—C4—C10—C9	0.2 (3)	C8A—C9A—C10A—C4A	179.3 (2)
N1—C9—C10—C5	179.1 (2)	C6A—C5A—C10A—C9A	0.7 (3)
C8—C9—C10—C5	−1.4 (3)	C6A—C5A—C10A—C4A	−178.9 (2)
N1—C9—C10—C4	−0.8 (3)	C3A—C4A—C10A—C9A	1.0 (3)
C8—C9—C10—C4	178.7 (2)	C3A—C4A—C10A—C5A	−179.5 (2)
C8—N2—C11—O1	−3.3 (4)	C8A—N2A—C11A—O1A	2.6 (4)
C8—N2—C11—C12	177.7 (2)	C8A—N2A—C11A—C12A	−176.5 (2)
O1—C11—C12—C13	−31.9 (3)	O1A—C11A—C12A—C13A	−27.5 (3)
N2—C11—C12—C13	147.2 (2)	N2A—C11A—C12A—C13A	151.7 (2)
O1—C11—C12—C17	147.3 (2)	O1A—C11A—C12A—C17A	152.8 (2)
N2—C11—C12—C17	−33.6 (3)	N2A—C11A—C12A—C17A	−28.0 (3)
C17—C12—C13—C14	1.5 (3)	C17A—C12A—C13A—C14A	0.7 (3)
C11—C12—C13—C14	−179.2 (2)	C11A—C12A—C13A—C14A	−179.0 (2)
C12—C13—C14—C15	−1.1 (3)	C12A—C13A—C14A—C15A	−1.8 (3)
C12—C13—C14—N3	178.03 (19)	C12A—C13A—C14A—N3A	177.4 (2)
O3—N3—C14—C15	−1.3 (3)	O3A—N3A—C14A—C13A	179.9 (2)
O2—N3—C14—C15	178.9 (2)	O2A—N3A—C14A—C13A	−0.8 (3)
O3—N3—C14—C13	179.5 (2)	O3A—N3A—C14A—C15A	−0.9 (3)
O2—N3—C14—C13	−0.2 (3)	O2A—N3A—C14A—C15A	178.4 (2)
C13—C14—C15—C16	−0.4 (3)	C13A—C14A—C15A—C16A	1.2 (4)
N3—C14—C15—C16	−179.57 (19)	N3A—C14A—C15A—C16A	−178.0 (2)
C14—C15—C16—C17	1.6 (3)	C14A—C15A—C16A—C17A	0.4 (3)
C15—C16—C17—C12	−1.2 (3)	C15A—C16A—C17A—C12A	−1.4 (3)
C13—C12—C17—C16	−0.4 (3)	C13A—C12A—C17A—C16A	0.8 (3)
C11—C12—C17—C16	−179.7 (2)	C11A—C12A—C17A—C16A	−179.5 (2)

### Hydrogen-bond geometry (Å, °)

**Table:**

*D*—H···*A*	*D*—H	H···*A*	*D*···*A*	*D*—H···*A*
C2—H2···O2^i^	0.95	2.56	3.395 (3)	147
C2A—H2A···O2^i^	0.95	2.50	3.346 (3)	148
C4—H4···O2A^i^	0.95	2.48	3.317 (3)	146
C16—H16···O3A^ii^	0.95	2.46	3.147 (3)	130
C16A—H16A···O1^iii^	0.95	2.50	3.198 (3)	130
C17—H17···O1A^iv^	0.95	2.45	3.396 (3)	172

Symmetry codes: (i) *x*+1, *y*, *z*+1; (ii) −*x*+1, *y*+1/2, −*z*+1; (iii) *x*+1, *y*, *z*; (iv) *x*, *y*, *z*+1.
